# State and Trait Anxiety Among University Students: A Moderated Mediation Model of Negative Affectivity, Alexithymia, and Housing Conditions

**DOI:** 10.3389/fpsyg.2020.01255

**Published:** 2020-06-10

**Authors:** Isabella Giulia Franzoi, Maria Domenica Sauta, Antonella Granieri

**Affiliations:** Department of Psychology, University of Turin, Turin, Italy

**Keywords:** university students, emerging adulthood, distress, negative affectivity, emotion, anxiety, depression, housing conditions

## Abstract

**Objective:**

Starting university education is a crucial period for the mental health of students, who report higher levels of distress compared to the general population. This study sought to better understand the distress experienced by students by considering contextual facets (e.g., housing conditions) as well as stable clinical variables (e.g., negative affectivity, emotion regulation, and anxiety).

**Methods:**

A total of 177 University students (71.2% females) aged 18-29 were administered the State-Trait Anxiety Inventory-Y, the Beck Depression Inventory-II, the Suicidal History Self-Rating Screening Scale, the Personality Inventory for DSM-5-Brief Form, and the Toronto Alexithymia Scale-20.

**Results:**

University students showed concerning levels of distress, particularly concerning anxiety, and depression. We found that the relationship between negative affectivity and both state and trait anxiety was mediated by alexithymia but housing conditions did not act as a moderator for the indirect effect of negative affectivity on state or trait anxiety through alexithymia.

**Conclusion:**

Undoubtedly, university lifestyle can be demanding, but experiencing distress is not inevitable nor inexplicable. The present study sought to gain insight into the anxiety experienced by Italian University students while taking into account the importance of personality and clinical characteristics that have previously been widely underestimated. We found that these characteristics can be of extreme importance for developing preventative and therapeutic interventions tailored to the clinical characteristics of students, as well taking into account their living environment.

## Introduction

### University Students’ Distress

Starting university is a challenging task for many young women and men in the transitional age between adolescence and adulthood, typically around 20 years of age. Not only do students face transformations connected to the emerging adulthood (Arnett, 2000; Arnett et al., 2014) such as emancipation, financial self-sufficiency, choices about career, and intimate relationships; they also must deal with further tasks connected to entering higher education such as relocation, performance demands, changes in living conditions and lifestyles, and dealing with a social and educational context far from the ones experienced before (Settersten and Ray, 2010; Schulenberg and Schoon, 2012).

Starting university seems to constitute a crucial period for the mental health of students (Molina et al., 2012; Pedrelli et al., 2015; Auerbach et al., 2016; Harris, 2019), who consistently report higher levels of distress compared to the general population (Stallman, 2010; Dachew et al., 2015; James et al., 2017; Tariku et al., 2017; Mboya et al., 2020). Previous research shows that 19.2–32% of university students reports mental health problems and subsyndromal symptoms (Stallman, 2010; Abdolhossini and Shikhmohamadi, 2012; Auerbach et al., 2016). Moreover, 17.3–41.1% of them reports psychiatric distress (Macaskill, 2013; Oksanen et al., 2017; Poorolajal et al., 2017). These data must be taken into account because the mental health of students has major implications for campus health services and mental health policymaking (Viñas et al., 2004).

Previous research has indicated high levels of depression, anxiety, and risk of suicide in students (see Ibrahim et al., 2013; Beiter et al., 2015; Larcombe et al., 2016; Rotenstein et al., 2016; Oyekcin et al., 2017; Poorolajal et al., 2017; Tran et al., 2017; Villatte et al., 2017; Tang et al., 2018).

### Students’ Distress and Housing Conditions

A major issue to take into account while considering the distress experienced by university students is that of housing conditions and the related daily routines. Indeed, housing has been identified as one of the main domains relating to individual well-being (van Praag et al., 2003; Sotgiu et al., 2011). In particular, housing overcrowding has negative associations with perceived housing quality, suggesting that the living space available for each occupant and the ability to control it play a fundamental role in subjective well-being (Caffaro et al., 2018).

University students living away from home or not owning the room they were living in showed higher psychological distress than students living at home or owning their room, regardless of their parental financial support (Stroebe et al., 2002; Vershuur et al., 2004; Flett et al., 2009; Watson et al., 2016). While separation from home does not necessarily have a negative impact, it may be a risk factor for vulnerable people who might experience an increase in depressive or anxiety symptoms and have a negative effect on their overall health (Thurber and Walton, 2012; Stroebe et al., 2015; Biasi et al., 2018).

### Affectivity, Emotion Regulation, and Anxiety

In exploring university students’ distress, we have to consider contextual facets such as housing conditions. As clinicians, we cannot underestimate the extent to which individuals who show high levels of negative affectivity generally manifest elevated levels of distress, anxiety, dissatisfaction, and a tendency toward focusing on the unpleasant aspects of themselves, other people, the world/life, and the future (Gross and Jazaieri, 2014; Jeronimus et al., 2014).

Affective experience can change across time and situations, but individuals tend toward some degree of stability. In particular, negative affectivity is a personality dimension (American Psychiatric Association, 2013) that develops early in life, although it can also be shaped by further experiences (Watson and Naragon-Gainey, 2014). It can be defined as the proneness to experience negative emotional states, and to activate defensive motivational systems (Craske, 2003). This leads to the tendency to frequently experience negative affective states (e.g., fear, sadness, anger, and guilt), to withdraw from potentially risky situations, and to react intensely to stress (Naragon-Gainey et al., 2018). Even if there is a certain association between negative affectivity and anxiety, these two constructs are not completely overlapping. Indeed, anxiety is an emotion characterized by an unpleasant state of inner turmoil, often accompanied by nervous behavior, somatic complaints, and rumination (Seligman et al., 2000). Anxiety is a feeling of uneasiness and worry, usually generalized and unfocused as an overreaction to a situation that is only subjectively seen as menacing (Bouras and Holt, 2007). Anxiety is often accompanied by muscular tension, restlessness, fatigue, and difficulties in concentration (American Psychiatric Association, 2013). Negative affectivity is the temperamental factor most commonly associated with anxiety and other emotional disorders (Lonigan and Phillips, 2001; Muris and Ollendick, 2005; Nigg, 2006). However, many individuals with heightened negative affectivity do not exhibit high levels of anxiety or develop anxiety disorders. Such findings have led researchers to examine the potential factors that mediate or moderate the relationship between affectivity and anxiety (Tortella-Feliu et al., 2010).

Emotion regulation and, more specifically, alexithymia are the constructs most frequently cited as playing a mediating role in the relationship between negative affectivity and anxiety. Indeed, research suggests that negative affectivity increases alexithymia (Bonnet et al., 2012; Gaher et al., 2015; Suslow and Donges, 2017). Even if affects and related personality dimensions have a pivotal impact on psychological distress, when we consider such links between affect and psychopathology, we also have to take into account the emotion regulation strategies that individuals activate to manage the feelings they are experiencing and to deal with distress (Sheppes et al., 2015). As suggested by Bagby et al. (1994), alexithymia can be described as a difficulty in identifying and describing feelings, as well as in distinguishing feelings from the bodily sensations of emotional arousal. Alexithymic individuals also exhibit constricted imaginative processes and externally oriented thinking (Taylor, 2000). They are often assailed by widespread negative affect, social evasion and poor emotional relationships with other people. From a wider clinical perspective, there is strong evidence that emotion regulation is closely related to most, if not all, anxious and depressive disorders. A positive association between emotional regulation and anxiety has been found, in particular between alexithymia (most notably difficulties identifying and describing feelings) and anxiety (Devine et al., 1999; Craske, 2003). The link between alexithymia and psychological distress has already been explored in university students, most notably relating to the symptoms of depression and neuroticism (Morera et al., 2005; Liang and West, 2011), self-injurious behaviors (Paivio and McCulloch, 2004), and interpersonal problems (Vanheule et al., 2007).

### Present Study

As far as we know, no study to date has investigated the relationship between negative affectivity, emotion regulation, and students’ state and trait anxiety while taking into account their housing conditions.

**FIGURE 1:**
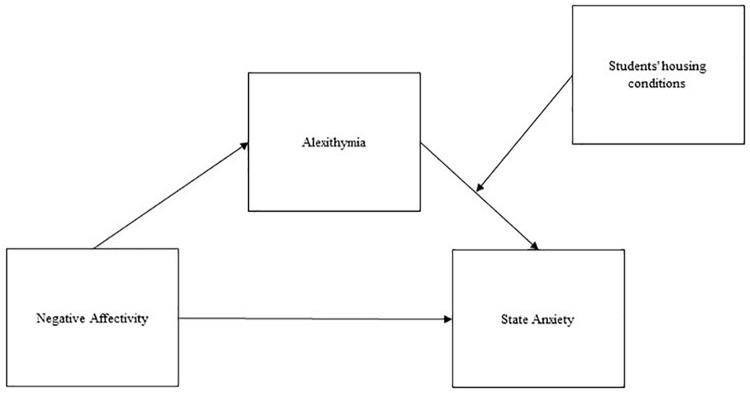
Diagram for the hypothesized moderated mediation model: state anxiety.

**FIGURE 2:**
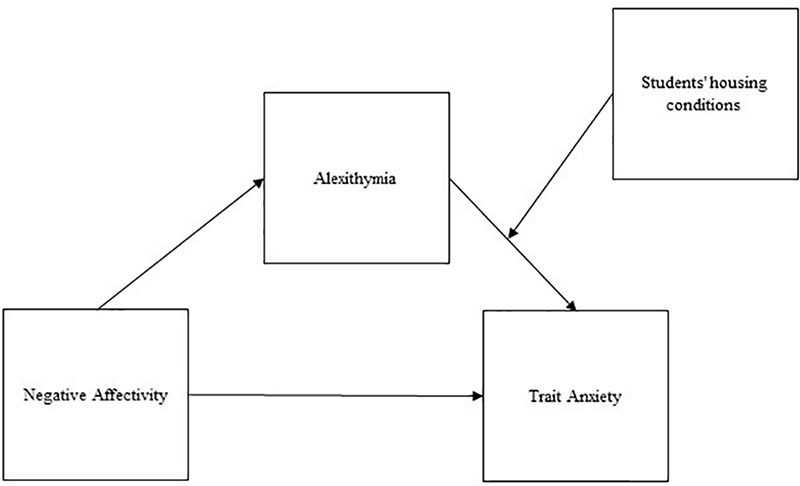
Diagram for the hypothesized moderated mediation model: trait anxiety.

We hypothesized that: (1) university students show high levels of distress, specifically anxiety (1a), depression (1b), and suicidal risk (1c); (2) students’ state and trait anxiety is connected with other clinical features such as negative affectivity (2a) and alexithymia (2b), but also with contextual facets such as housing conditions (2c). We also hypothesized that alexithymia mediates the relationship between negative affectivity and anxiety. Previous research has already suggested this mediation effect, but as far as we know the model has not yet been tested on university students. In addition, since housing conditions have already been linked to anxiety by previous research, we decided to include them in our model as a moderator. Indeed, literature shows how alexithymia and negative affectivity are rarely, or only minimally, influenced by contextual variables (Mroczek and Kolarz, 1998; Luminet et al., 2007). Thus, our final hypothesis (3) was that. the relationship between negative affectivity and anxiety is mediated by alexithymia, while housing conditions act as a moderator for the indirect effect of negative affectivity on anxiety through alexithymia. Our moderated mediation models tested are represented in Figure 1 (for state anxiety) and Figure 2 (for trait anxiety).

## Materials and Methods

### Study Design and Participants

The present research was a descriptive, cross-sectional study. It was part of a wider study investigating psychological distress and housing conditions among University of Turin (UniTo) students. The wider project included purposive sampling (Corbetta,2015a, b) based on four housing conditions: students living with their family of origin, commuter students, non-resident students living in a university residence, and non-resident students not living in a residence. For each housing condition, the sampling target was to include an equal number of students attending courses pertaining to the three European Research Council (ERC) research domains: Social Sciences and Humanities (SH), Physical sciences and Engineering (PE), and Life Sciences (LS). This project is still ongoing. In the current research, we considered students’ housing conditions by dividing them into two groups: resident students (students living with their family of origin and commuter students) and non-resident students (regardless of whether they lived in a university residence). Since the recruitment for the wider project is not yet concluded, for the present project we could not include an equal number of students for each of the three ERC research domains.

Students were recruited between October 2018 and December 2019.

Inclusion criteria were being students aged between 18 and 29 years (emerging adults) and attending UniTo Courses.

Exclusion criteria were having poor knowledge of the Italian language, being aged more than 29 years old and attending another tertiary education institution.

The enrollment was conducted through cooperation with professors, student representatives, departmental councils, and Heads of Departments. We reached 28 (41.8%) out of the 67 bachelor’s degree courses and three (33.3%) out of the nine single-cycle master’s degree courses available in UniTo. We emailed 128 professors, and 46 (35.93%) gave their availability to host a research presentation in their class. We had positive responses from 32 (25.0%) SH professors, 11 (8.60%) PE professors, and only three (7.03%) LS professors. Eight hundred and sixty-nine students declared their willingness to participate in the study. Among these, only 177 (20.36%) students agreed to be tested.

### Outcome Measures

The complete study included the administration of a questionnaire aimed at investigating socio-demographic characteristics and a pool of self-report questionnaires validated for the Italian population. In the present research, we consider only socio-demographic data and the scores obtained from the State-Trait Anxiety Inventory-Y (STAI-Y; Spielberger et al., 1983; Pedrabissi and Santinello, 1996), the Beck Depression Inventory-II (BDI-II; Beck et al., 1996; Ghisi et al., 2006), the Suicidal History Self-Rating Screening Scale (SHSS; Innamorati et al., 2011), the Personality Inventory for DSM-5-Brief Form (PID-5-BF; Fossati et al., 2013; Krueger et al., 2013), and the Toronto Alexithymia Scale-20 (TAS-20; Bagby et al., 1994; Bressi et al., 1996).

The administration was conducted at the university in the presence of a psychologist or a trained post-graduate psychology student. The average time of completion was 39.80 ± 9.86 min (range 20–77 min).

The STAI-Y is a 40-item self-report inventory aimed at assessing two types of anxiety symptoms: state anxiety (i.e., how a person in the current situation responds to perceived threat) and trait anxiety (i.e., the stable tendency to attend, experience, and report negative emotions such as fears, worries, and anxiety across many situations). Each of these dimensions comprises 20 non-overlapping trait facets. Participants are asked to rate how accurately each of the items describe them on a five-point scale (ranging from 1 = “almost never” to 4 = “almost always”). As suggested by previous research (e.g., Hart and McMahon, 2006; El Sawy, 2012), we used a cut-off of 40 to evaluate the presence or absence of state and trait anxiety. STAI-Y original version demonstrates good internal consistency (Cronbach’s alpha = 0.90) and test-retest reliability (*r* = 0.70, *p* < 0.001). For the Italian version, both state and trait scales demonstrate good internal consistency (Cronbach’s alpha = 0.93 and 0.88, respectively) and test-retest reliability (*r* = 0.49 and 0.82, respectively).

The BDI-II is a 21-item self-report questionnaire in which each item corresponds to a specific category of depressive symptoms and attitudes. Participants are asked to choose between four options for each item ranging from 0 to 3 (0 = “I do not feel sad”; 3 = “I am so sad or unhappy that I can’t stand it”). Higher total scores indicate more severe depressive symptoms. Scores from 14 to 19 indicate mild depression, scores from 20 to 28 indicate moderate depression, and scores from 29 to 63 indicate severe depression. BDI-II demonstrates good internal consistency and test-retest reliability both in the original version (Cronbach’s alpha = 0.91; *r* = 0.93, *p* < 0.001), and in the Italian sample (Cronbach’s alpha = 0.90; *r* = 0.85; *p* < 0.001).

The SHSS is a 16-item measure assessing thoughts of death, suicidal ideation, and behavior. Participants are asked to answer eight yes/no questions concerning the last 12 months and eight yes/no questions concerning their lifetime except for the last 12 months. Higher total scores indicate more severe suicidal ideation, and scores > 8 indicate a risk for suicidal behavior. The SHSS was specifically developed and validated for Italian samples and demonstrates good internal consistency (Cronbach’s alpha = 0.80).

The TAS-20 is a 20-item self-reported measure of alexithymia. It has a three-factor structure: Difficulty in Identifying Feelings (DIF), Difficulty in Describing Feelings (DDF), and Externally Oriented Thinking (EOT). A TAS-20 total score ≥ 61 is considered indicative of alexithymia, whereas scores between 51 and 60 indicate borderline alexithymia. Participants are asked to rate how accurately each of the items describe them on a five-point scale (ranging from 1 = “completely disagree” to 5 = “completely agree”). TAS- 20 demonstrates good internal consistency and test-retest reliability both in the original version (Cronbach’s alpha = 0.81; *r* = 0.77, *p* < 0.001), and in the Italian validation (Cronbach’s alpha = 0.82; *r* = 0.86; *p* < 0.001).

The PID-5-BF is a 25-item dimensional self-report measure assessing five broad pathological personality traits: Negative Affectivity (NA), Detachment (DE), Antagonism (A), Disinhibition (DI), and Psychoticism (P). Each of these five higher-order dimensions comprises five non-overlapping trait facets. Participants are asked to rate how accurately each item describes them on a four-point scale (ranging from 0 = “very false or often false” to 3 = “very true or often true”). The PID-5 demonstrates good internal consistency in Italian samples, with Cronbach’s alpha values > 0.93 for all domain scales.

### Statistical Analyses

Data analyses were conducted using the Statistical Package for the Social Sciences (SPSS; IBM Corp., Armonk, NY, United States) version 26. We calculated descriptive statistics and χ^2^-tests to get a preliminary description of the sociodemographic and clinical characteristics of the sample. Then we conducted Spearman, point-biserial, and Pearson correlations to get an initial overview of the variables to be included in our moderated mediation model. All tests were two-tailed, and we set the statistical significance threshold at p ≤ 0.05. Finally, we conducted two moderated mediation analyses (one for State Anxiety and one for Trait Anxiety) using the PROCESS macro for SPSS (version 3.4.1; Hayes, 2018) using model 14. The direct and indirect effects were estimated using the Preacher and Hayes (2004) bias-corrected non-parametric bootstrapping techniques with 5,000 bootstrap samples. We used the mean center for the construction of products. As suggested by prior research (Settanni et al., 2018; Jin et al., 2019), the existence of mediation and moderated mediation effects were further evaluated using 95% bias-corrected confidence intervals (CIs). If the CIs did not contain zero, these effects were considered statistically significant.

## Results

### Sociodemographic and Clinical Characteristics of the Sample

As shown in Table 1, our final sample consisted of 177 UniTo students (71.2% females) with a mean age of 21.54 (*SD* = 2.14). Regarding demographic data, 99.4% of the students were not married, 130 students came from Northern Italy (73.4%), 11 from Middle Italy (6.2%), and 33 from Southern Italy or the Islands (8.7%). In total, 65.5% of the sample were unemployed, 63.9% of them had a medium family income level, and 50.3% were non-resident students. As reported in Table 2, the sample was evenly distributed among ERC domains and housing conditions (χ^2^ = 1.042; *p* = 0.594), and among family income level and housing conditions (χ^2^ = 0.07; *p* = 0.967).

**TABLE 1:** Socio-demographic characteristics of the sample.

	n	%	
**Gender**			
Males	51	28.8	
Females	126	71.2	

	***M***	***SD***	**Range**

Age	21.54	2.14	18–29

	**n**	**%**	

**Marital status**			
Not married	176	99.4	
Other	1	0.6	
**Geographic origin**			
Northern Italy	130	73.4	
Middle Italy	11	6.2	
Southern Italy	24	13.6	
Islands	9	5.1	
ND	3	1.7	
**Occupation**			
Unemployed	116	65.5	
Occasional worker	49	27.7	
Employee	11	6.2	
ND	1	0.6	
**Erc domains**			
SH	103	58.2	
PE	56	31.6	
LS	18	10.2	
**Housing conditions**			
Resident students	88	49.7	
Non-resident students	89	50.3	
**Family income level**			
Low	35	19.8	
Medium	113	63.9	
High	29	16.4	

**TABLE 2:** χ^2^-tests.

	Resident students (*n* = 88)		Non-resident students (*n* = 89)			
	n	%	n	%	χ^2^	*p*
ERC domains					1.042	0.594
SH	50	56.8	53	59.6		
PE	27	30.7	29	32.6		
LS	11	12.5	7	7.9		
**Family income level**					0.070	0.967
Low	17	19.3	18	20.2		
Medium	56	63.6	57	64.0		
High	15	17.0	14	15.7		

Concerning psychological distress (Table 3), UniTo students showed state (*M* = 40.67; *SD* = 12.35) and trait (*M* = 45.28; *SD* = 11.63) anxiety. More specifically, 44.1% showed state anxiety, 61.6% showed trait anxiety, and 40.1% showed both. Both state and trait anxiety were normally distributed with a skewness of 0.66 (*SE* = 0.18) and 0.33 (*SE* = 0.18), respectively, and a kurtosis of −0.17 (*SE* = 0.36) and −0.61 (*SE* = 0.36), respectively. Our sample also showed minimal depressive symptoms (*M* = 12.4; *SD* = 10.17) with moderate to severe depression in 20.4% of cases. Depression was not normally distributed, with a skewness of 1.26 (*SE* = 0.18) and a kurtosis of 1.28 (*SE* = 0.36). SHSS shows a not-at-risk mean value (*M* = 1.94; *SD* = 2.89). However, 3.4% of the sample were at risk for suicidal behaviors. Suicidal risk was not normally distributed, with a skewness of 1.67 (*SE* = 0.18) and a kurtosis of 2.19 (*SE* = 0.36).

**TABLE 3:** Clinical characteristics of the sample.

	*M*	*SD*		Range		Skewness		Kurtosis	
						Statistic	Std. Error	Statistic	Std. Error
STAI-Y state	40.67	12.35		20–76		0.66	0.18	–0.17	0.36
STAI-Y trait	45.28	11.63		23–73		0.33	0.18	–0.61	0.36
SHSS TOT	1.94	2.89		0–13		1.67	0.18	2.19	0.36
BDI-II TOT	12.4	10.17		0–47		1.26	0.18	1.28	0.36
TAS-20 TOT	47.93	12.49		21–85		0.12	0.18	–0.39	0.36
PID-5-BF-NA	1.38	0.63		0–3.2		0.10	0.18	–0.14	0.36
PID-5-BF-DE	0.75	0.57		0–2.4		0.60	0.18	–0.46	0.36
PID-5-BF-A	0.52	0.44		0–2.0		1.04	0.18	1.14	0.36
PID-5-BF-DI	0.85	0.5		0–2.4		0.49	0.18	0.12	0.36
PID-5-BF-P	0.75	0.67		0–3.0		0.93	0.18	0.48	0.36
PID-5-BF-TOT	0.88	0.47		0.12–0.46		2.89	0.18	21.47	0.36

	**n**	**%**							

**STAI-Y STATE LIV**									
No state anxiety	99	55.9							
State anxiety	78	44.1							
**STAI-Y TRAIT LIV**									
No trait anxiety	68	38.4							
Trait anxiety	109	61.6							
**BDI-II LIV**									
Minimal depression	114	64.4							
Mild depression	27	15.3							
Moderate depression	21	11.9							
Severe depression	15	8.5							
**SHSS RISK**									
No	171	96.6							
Yes	6	3.4							
**TAS-20 LIV**									
Alexithymia	28	15.8							
Borderline alexithymia	55	31.1							
No alexithymia	94	53.1							

		**Trait anxiety**							
		**No trait anxiety**		**Trait anxiety**					
		**n**	**% tot**	**n**	**% tot**				

State anxiety	No state anxiety	61	34.5	38	21.5				
	State anxiety	7	4.0	71	40.1				

Regarding the other clinical characteristics of the sample (Table 3), students’ mean scores suggest an overall absence of alexithymia (*M* = 47.93, *SD* = 12.49). However, 15.8% showed alexithymia, and 31.1% had borderline scores. Alexithymia was normally distributed, with a skewness of 0.12 (*SE* = 0.18) and a kurtosis of −0.39 (*SE* = 0.36). The level of personality impairment was mild (*M* = 0.88; *SD* = 0.47). Students were characterized by mild levels of negative affectivity (*M* = 1.38; *SD* = 0.63), psychoticism (*M* = 0.75; *SD* = 0.67), detachment (*M* = 0.75; *SD* = 0.57), disinhibition (*M* = 0.85; *SD* = 0.67), and antagonism (*M* = 0.52; *SD* = 0.44). The level of personality distress was not normally distributed, with a skewness of 2.89 (*SE* = 0.18) and a kurtosis of 21.47 (*SE* = 0.36), as well as antagonism, with a skewness of 1.04 (*SE* = 0.18) and a kurtosis of 1.14 (*SE* = 0.36). However, negative affectivity, detachment, disinhibition and psychoticism, with a skewness of 0.10 (*SE* = 0.18), 0.60 (*SE* = 0.18), 0.49 (*SE* = 0.18), and 0.93 (*SE* = 0.18), respectively, and a kurtosis of -0.14 (*SE* = 0.36), -0.46 (*SE* = 0.18), 0.12 (*SE* = 0.18), and 0.48 (*SE* = 0.36), respectively.

### Preliminary Analyses for the Moderated Mediation Model

As expected (see Table 4), negative affectivity correlated positively with alexithymia (*r* = 0.432; *p* < 0.001), state anxiety (*r* = 0.505; *p* < 0.001), and trait anxiety (*r* = 0.675; *p* < 0.001). At the same time, alexithymia showed a positive and significant correlation with state anxiety (*r* = 0.414; *p* < 0.001) and trait anxiety (*r* = 0.563; *p* < 0.001). In terms of the hypothesized covariates, negative affectivity correlated positively with gender (*r* = 0.188; *p* = 0.012) and negatively with age (*r* = −0.227; *p* = 0.002), alexithymia correlated negatively with age (*r* = −0.344; *p* < 0.001), and family income level positively correlated only with age (*r* = 0.186; *p* = 0.013). Thus, in our final moderated mediation model, we decided to include only age and gender as covariates. Unexpectedly, housing conditions showed a significant correlation only with negative affectivity (*r* = −0.154; *p* = 0.041).

**TABLE 4:** Pearson, point-biserial, and Spearman correlations.

	TAS-20 TOT	STAI-Y state	STAI-Y trait	PID AN	Age	Gender	Housing conditions
**Pearson’s correlations**							
STAI-Y State	0.414**						
STAI-Y Trait	0.563**	0.727**					
PID AN	0.432**	0.505**	0.675**				
Age	−0.344**	–0.135	−0.208**	−0.227**			
**Point-biserial correlations**							
Gender	–0.038	0.090	0.139	0.188*	−0.160*		
Housing conditions	–0.116	–0.014	–0.036	−0.154*	0.131	–0.084	
**Spearman’s correlations**							
Family income level	0.011	0.008	–0.036	–0.067	0.186*	0.005	–0.018

** p < 0.01, * p < 0.05.

### Moderated Mediation Analysis

The first regression analysis showed a significative positive effect of negative affectivity on alexithymia (β = 7.96, *SE* = 1.35, *p* < 0.001. We also found a negative and significant effect of gender (β = −4.32, *SE* = 1.83, *p* = 0.019) and age (β = −1.63, *SE* = 0.39, *p* < 0.001) on alexithymia. Altogether, the predictors explained 27% of the variance observed in alexithymia scores [*F*(3, 173) = 21.75, *p* < 0.001; see Table 5].

**TABLE 5:** Moderated mediation model analysis: first step.

Outcome variables	Independent variables	β	SE	*t*	*p*	95%CI
**TAS-20-TOT**						
	Constant	27.13	9.23	2.94	0.004	[8.91;45.35]
	PID_AN_M	7.96	1.35	5.92	<0.001	[5.31;10.62]
	Gender	−4.32	1.83	−2.36	0.019	[−7.92;−0.71]
	Age	−1.63	0.39	−4.15	<0.001	[−2.40;−0.85]

#### State Anxiety

As shown in Table 6, the second regression analysis showed a positive and significant effect of negative affectivity on state anxiety (β = 8.02, *SE* = 1.44, *p* < 0.001) and of alexithymia on state anxiety (β = 0.26, *SE* = 0.07, *p* < 0.001). However, student housing conditions was not a significant predictor of state anxiety (β = 1.88, *SE* = 1.60, *p* = 0.240). Concerning the covariates, neither gender (β = 0.92, *SE* = 1.81, *p* = 0.614) nor age (β = 0.24, *SE* = 0.40, *p* = 0.549) were significant predictors of state anxiety. The indirect effect of alexithymia on state anxiety was not significant (β = 0.07, *SE* = 0.13, *p* = 0.593). Overall, the predictors explained 31% of the variance observed in state anxiety [*F*(6, 170) = 12.84, *p* < 0.001]. The inclusion of the interaction between alexithymia and housing conditions in the regression model led to a change in *R*^2^ = 0.001 [*F*(1, 170) = 0.29, *p* = 0.593].

**TABLE 6:** Moderated mediation model analysis: state anxiety.

Outcome variables	Independent variables	β	*SE*	*t*	*p*	95%CI
STAI-Y state	Constant	23.85	9.22	2.59	0.010	[5.65;42.05]
	PID_AN_M	80.2	1.44	5.57		[5.17;10.86]
	TAS-20 TOT	0.26	0.07	3.49		[5.18:10.86]
	Housing conditions	1.88	1.60	1.18	0.240	[−1.27;5.04]
	Int_1	0.07	0.13	0.54	0.593	[−0.184;0.320]
	Gender	0.92	1.82	0.51	0.61	[−2.67;4.50]
	Age	0.24	0.40	0.60	0.549	[−0.55;1.03]

**FIGURE 3:**
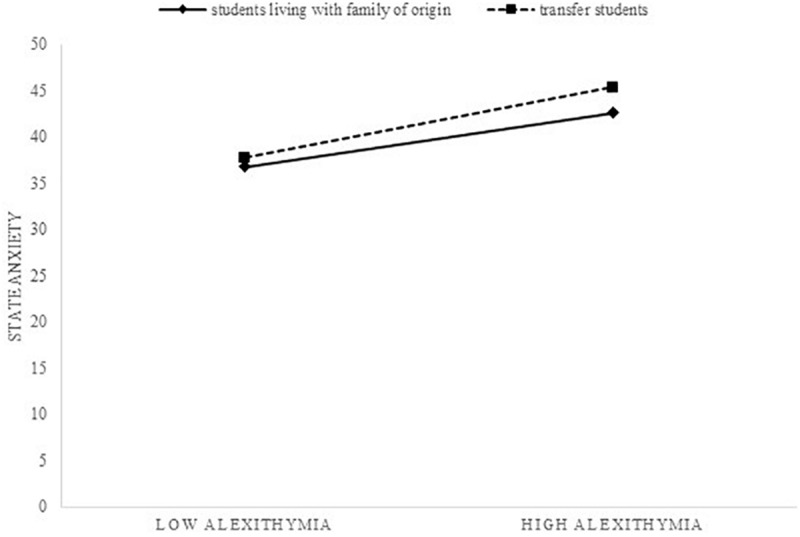
Simple slope analysis for stale anxiety.

The simple slope analysis (see Figure 3) of the interaction model showed a significant positive relationship between alexithymia and state anxiety for both resident (β = 0.22, *SE* = 0.10, *p* = 0.028) and non-resident students (β = 0.29, *SE* = 0.10, *p* = 0.002). For resident students, the moderated mediation model was significant (β = 1.78, bootstrap *SE* = 0.93, bootstrap 95% CI = 0.02; 3.71) as well as for non-resident students (β = 2.33, bootstrap *SE* = 0.93, bootstrap 95% CI = 0.81; 4.46). Overall, the moderated mediation model was not significant (β = 0.55, bootstrap *SE* = 1.11, bootstrap 95% CI = -1.48; 2.91; see Table 7).

**TABLE 7:** Direct and indirect effects of study variables: state anxiety.

	β	SE	95%CI
**Direct effect of negative affectivity on state anxiety**			
	8.02	1.4	[5.18;10.86]

	**β**	**Bootstrap *SE***	**Bootstrap 95%CI**

**Conditional indirect effects of negative affectivity on state anxiety via alexithymia at different housing conditions**			
Resident students	1.78	0.93	[0.02;3.71]
Non-resident students	2.33	0.93	[0.81;4.46]
**Index of moderated mediation**			
	0.55	1.1	[1.48: 2.91]

#### Trait Anxiety

As shown in Table 8, the second regression analysis shows a positive and significant effect of negative affectivity on trait anxiety (β = 9.84, *SE* = 1.08, *p* < 0.001) and alexithymia on trait anxiety (β = 0.34, *SE* = 0.06, *p* < 0.001). On the contrary, student housing conditions was not a significant predictor of trait anxiety (β = 2.07, *SE* = 1.20, *p* = 0.086). Concerning our covariates, neither gender (β = 1.90, *SE* = 1.36, *p* = 0.166) nor age (β = 0.21, *SE* = 0.30, *p* = 0.488) were significant predictors of trait anxiety. The indirect effect of alexithymia on trait anxiety was not significant (β = −0.14, *SE* = 0.10, *p* = 0.149). Overall, the predictors explained 56% of the variance observed in trait anxiety [*F*(6, 170) = 36.58, *p* < 0.001]. The inclusion of the interaction between alexithymia and housing conditions in the regression model led to a change in *R*^2^ = 0.006 [*F*(1, 170) = 2.10, *p* = 0.149].

**TABLE 8:** Moderated mediation model analysis: trait anxiety.

Outcome variables	Independent variables	β	*SE*	*T*	*p*	95%CI
STAI-Y trait	Constant	25.79	6.92	3.73	<0.001	[12.14;39.44]
	PID_AN_M	9.84	1.08	9.12	<0.001	[7.71;11.97]
	TAS-20 TOT	0.34	0.06	6.12	<0.001	[0.23;0.45]
	Housing conditions	2.07	1.20	1.73	0.086	[-0.29;4.43]
	Int_1	−0.14	0.10	−1.45	0.149	[−0.33;0.05]
	Gender	1.90	1.36	1.39	0.166	[−0.79;4.58]
	Age	0.21	0.30	0.70	0.488	[-0.38;0.80]

**FIGURE 4:**
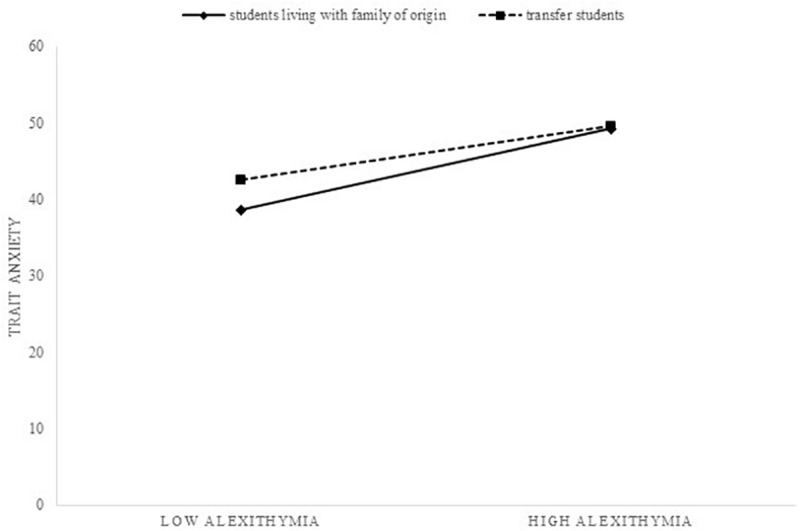
Simple slope analysis for trail anxiety.

The simple slope analysis (see Figure 4) of the interaction model showed a significant positive relationship between alexithymia and trait anxiety for both resident (β = 0.41, *SE* = 0.08, *p* < 0.001) and non-resident students (β = 0.27, *SE* = 0.07, *p* < 0.001). For students living with their family of origin, the model was significant (β = 3.26, bootstrap *SE* = 0.77, bootstrap 95% CI = 1.88; 4.90) as well as for non-resident students (β = 2.15, bootstrap *SE* = 0.72, bootstrap 95% CI = 0.98; 3.79). Overall, the moderated mediation model was not significant (β = −1.11, bootstrap *SE* = 0.78, bootstrap 95% CI = −2.61; 0.53; see Table 9).

**TABLE 9:** Direct and indirect effects of study variables: trait anxiety.

	β	*SE*	95%CI
**Direct effect of negative affectivity on trait anxiety**			
	9.84	1.08	[7.71;11.97]

	**β**	**Bootstrap *SE***	**Bootstrap 95%CI**

**Conditional indirect effects of negative affectivity on trait anxiety via alexithymia at different housing conditions**			
Resident students	3.26	0.77	[1.88;4.90]
Non-resident students	2.15	0.72	[0.98;3.79]
**Index of moderated mediation**			
	−1.11	0.78	[−2.61;0.53]

## Discussion

This study investigated university students’ distress, and in particular considered the potential relationship between negative affectivity, emotion regulation, and students’ anxiety, taking into account their housing conditions.

Consistent with previous studies from other European universities (Oksanen et al., 2017; Shankland et al., 2018; Çebi and Demir, 2019; Piumatti et al., 2019; Véron et al., 2019), UniTo students showed concerning levels of distress, confirming our first hypothesis.

In particular, UniTo students showed higher levels of both trait and state anxiety (hypothesis 1a) compared with adult workers and high school students in the Italian normative sample (Pedrabissi and Santinello, 1996). However, to our knowledge there are no normative Italian data on emerging adults. The percentages of students showing state anxiety, trait anxiety, or both are higher than those detected in other studies, although they relied on clinical data (Tran et al., 2017) or other self-report questionnaires (Ozen et al., 2010; Wörfel et al., 2016; Oyekcin et al., 2017).

Concerning depression (hypothesis 1b), our sample showed minimal depressive symptoms, as has other university student samples (Chen et al., 2013; Chun et al., 2013; Reyes-Rodríguez et al., 2013; Villatte et al., 2017). Consistent with previous literature, the prevalence of moderate and severe depression among students is remarkable (Chen et al., 2013; Villatte et al., 2017).

Regarding the prevalence of both anxiety and depression, it would be interesting to replicate this study in other Italian universities and to evaluate the evolution of symptoms over time, since previous research suggests that their prevalence differs in different class years (Wörfel et al., 2016).

Regarding suicide (hypothesis 1c), our data indicate a lower suicidal risk than that detected in previous studies on university students, although this discrepancy could be attributable to the different outcome measures (Chesin and Jeglic, 2012; Oyekcin et al., 2017; Poorolajal et al., 2017; Torres et al., 2017). However, we cannot underestimate the risk for suicidal behaviors in 3.4% of the sample.

Regarding the other clinical characteristics of the sample, consistent with the literature, our data indicated a prevalence of no severe personality disorders in the student sample (Duroy et al., 2018; Abdi and Pak, 2019). Concerning alexithymia, as expected, students’ mean scores also suggest no alexithymia, although a notable percentage of students showed alexithymic or borderline scores, indicating some difficulties in the emotion regulation process (Fang et al., 2019; Loftis et al., 2019).

Moving on to our second hypothesis, consistent with the literature (Picardi et al., 2005; Hofman et al., 2019), students’ state and trait anxiety were positively connected with other clinical variables – negative affectivity (hypothesis 2a) and alexithymia (hypothesis 2b). Concerning the hypothesized covariates, consistent with the literature, negative affectivity was higher in females and younger students (Ortuno-Sierra et al., 2019; Elhai et al., 2020), while alexithymia was higher in younger students (Mattila et al., 2006; Moriguchi et al., 2007).

Contrary to the existing literature (Scimeca et al., 2014; Hamaideh, 2018), no association was found between alexithymia and gender. Unexpectedly, family income level was correlated only with age (Song and Kim, 2020). Thus, in our final moderated mediation model, we included only age and gender as covariates.

In relation to the contextual variables (i.e., housing conditions; hypothesis 2b), contrary to previous research, we did not find a significant correlation with student anxiety (Stroebe et al., 2015; Biasi et al., 2018). However, although housing conditions did not seem to be associated with alexithymia and anxiety, we proceeded with testing our moderated mediation model to explore whether they had an impact on the association or whether the connection between negative affectivity, alexithymia, and anxiety differed in the two housing conditions.

Concerning our moderated mediation models, in line with our third hypothesis, we found a significant positive effect of negative affectivity on alexithymia while controlling for age and gender. This is in line with previous research indicating that greater levels of negative affectivity are associated with greater alexithymia (Suslow and Donges, 2017) and suggesting that the manner in which emotions are experienced determines, to some extent, the ability to regulate emotions and the degree to which one attempts to control and avoid them (Lynch et al., 2001; Suveg et al., 2009).

Concerning anxiety, the findings supported our hypothesis that alexithymia mediates the association between negative affectivity and both state and trait anxiety while controlling for age and gender. Such findings are compatible with prior research indicating that negative affectivity is positively associated with emotion regulation strategies (Naragon-Gainey et al., 2018; Malesza, 2019) and that emotion regulation strategies can help with modulating anxiety (Craske, 2003; Lonigan and Vasey, 2009). The current study brings these facets together, demonstrating the pathway from negative affectivity to state and trait anxiety via alexithymia. However, the results did not confirm our hypothesis that students’ housing conditions have a significant impact on anxiety. For both resident and non-resident students, students with lower alexithymia are characterized by lower anxiety.

Further research is needed to explore whether housing conditions are a significant factor relating to student anxiety in UniTo students and also in other Italian samples. Moreover, it would be interesting to explore whether differences can be observed if we consider not only resident vs. non-resident students but more specific housing conditions such as those of students living with their family of origin, commuter students, non-resident students in university residences, and other non-resident students.

In a time when educational systems all over the world have recently increased their concern for the mental health and emotional well-being of university students (Cvetkovski et al., 2018), our results suggest the importance of stable clinical variables in students’ distress and of not only focusing on contextual facets of their daily lives.

Undoubtedly, university lifestyle can be demanding, but experiencing distress is not inevitable nor inexplicable. Previous research has tried to identify factors associated with university students’ distress, aiming at using them to inform prevention and clinical interventions. However, as noted by Sharp and Theiler (2018), although socio-demographic, contextual, and academic variables have been widely explored, suggesting the need for interventions addressed to at-risk students, the importance of students’ personality and clinical characteristics has been underestimated. Nevertheless, these characteristics can be of extreme importance both in targeting interventions and in training health professionals who administer those interventions. Moreover, institutional practices and governmental policies that can influence the student experience need to be considered and deserve further consideration (Byrd and McKinney, 2012).

### Limitations and Future Directions

This study has some critical limitations. First of all, the generalizability of the results is limited by our small, Italian-only sample from only one university. Second, the cross-sectional design does not allow for causal inferences. For this reason, we should be cautious in interpreting the present findings as supporting the existence of predictive links between the studied variables. Further longitudinal studies are needed to explore the development of university students’ distress over time and its association with other clinical and social variables. Moreover, psychological variables were assessed through self-report measures and, as such, further studies should also consider clinical and observational data.

### Clinical Implications

Despite these limitations, the present study is the first attempt to obtain insight into Italian University students’ distress, focusing on state and trait anxiety and their connections with both clinical facets (i.e., negative affectivity and emotional regulation) and contextual facets (i.e., student housing conditions). The difficulties university students face are a matter of public concern. Thus, our results can be useful for both professional and clinical or educational institutions since it is well known that students experiencing higher psychological distress show a higher risk of academic failures and drop-out (Jaisoorya et al., 2017; Ishii et al., 2018). Such evidence strongly suggests the need to adopt an integrated approach toward university students to alleviate their psychological distress, and to improve the development of preventative and therapeutic interventions tailored to the clinical characteristics of students, as well as taking their living environment into account.

## Data Availability Statement

The datasets generated for this study are available on request to the corresponding author.

## Ethics Statement

The study was reviewed and approved by the Institutional Review Board (IRB) of the University of Turin (prot. n. 162317 of the 4/19/2018). All participants were given a complete description of the study and gave informed written consent before entering the study. All research procedures were conducted in accordance with the ethical standards of the committees responsible for human experimentation (institutional and national) and with the Helsinki Declaration of 1975 (as revised in 2000). The patients/participants provided their written informed consent to participate in this study.

## Author Contributions

IF contributed to the study design, the analysis and interpretation of data, the drafting and critical revision of the manuscript. MS contributed to the analysis and interpretation of data and drafting the manuscript. AG contributed to the interpretation of data, making an important clinical, and intellectual contribution. All authors approved the final version of the manuscript to be published and agreed to be accountable for all aspects of the work in ensuring that questions related to the accuracy and integrity of any part of the work were appropriately investigated and resolved.

## Conflict of Interest

The authors declare that the research was conducted in the absence of any commercial or financial relationships that could be construed as a potential conflict of interest.
